# Implementation of an Electronic Monitoring and Evaluation System for the Antiretroviral Treatment Programme in the Cape Winelands District, South Africa: A Qualitative Evaluation

**DOI:** 10.1371/journal.pone.0127223

**Published:** 2015-05-12

**Authors:** Hanlie Myburgh, Joshua P. Murphy, Mea van Huyssteen, Nicola Foster, Cornelius J. Grobbelaar, Helen E. Struthers, James A. McIntyre, Theunis Hurter, Remco P. H. Peters

**Affiliations:** 1 Anova Health Institute, Paarl, Western Cape Province, South Africa; 2 Anova Health Institute, Johannesburg, Gauteng Province, South Africa; 3 School of Pharmacy, Faculty of Natural Science, University of the Western Cape, Bellville, Western Cape Province, South Africa; 4 School of Public Health and Family Medicine, University of Cape Town, Cape Town, Western Cape Province, South Africa; Örebro University, SWEDEN

## Abstract

**Background:**

A pragmatic three-tiered approach to monitor the world’s largest antiretroviral treatment (ART) programme was adopted by the South African National Department of Health in 2010. With the rapid expansion of the programme, the limitations of the paper-based register (tier 1) were the catalyst for implementation of the stand-alone *electronic* register (tier 2), which offers simple digitisation of the paper-based register. This article engages with theory on implementation to identify and contextualise enabling and constraining factors for implementation of the electronic register, to describe experiences and use of the register, and to make recommendations for implementation in similar settings where standardisation of ART monitoring and evaluation has not been achieved.

**Methods:**

We conducted a qualitative evaluation of the roll-out of the register. This comprised twenty in-depth interviews with a diverse sample of stakeholders at facility, sub-district, and district levels of the health system. Facility-level participants were selected across five sub-districts, including one facility per sub-district. Responses were coded and analysed using a thematic approach. An implementation science framework guided interpretation of the data.

**Results & Discussion:**

We identified the following seven themes: 1) ease of implementation, 2) perceived value of an electronic M&E system, 3) importance of stakeholder engagement, 4) influence of a data champion, 5) operational and logistical factors, 6) workload and role clarity, and 7) importance of integrating the electronic register with routine facility monitoring and evaluation. Interpreting our findings through an implementation theory enabled us to construct the scaffolding for implementation across the five facility-settings. This approach illustrated that implementation was not a linear process but occurred at two nodes: at the adoption of the register for roll-out, and at implementation at facility-level.

**Conclusion:**

In this study we found that *relative advantage* of an intervention and stakeholder *engagement* are critical to implementation. We suggest that without these aspects of implementation, formative and summative outcomes of implementation at both the adoption and coalface stages of implementation would be negatively affected.

## Introduction

South Africa’s antiretroviral treatment (ART) programme is the largest in the world with more than two million patients on treatment in 2012. These patients represent almost a third of the approximately 6.4 million HIV-positive individuals in South Africa [1]. This programme has been rapidly evolving since initial roll-out to public sector health care facilities in 2004. With continued efforts to expand treatment coverage and on-going debates concerning earlier ART initiation, the scale-up of the programme necessitates a robust monitoring and evaluation (M&E) system able to signpost programme progress and performance [2] at a national level.

Monitoring and evaluation have played a key role in successful scale-up. Initially, the South African National Department of Health endorsed a vertical ART M&E system; however guidelines on the design and implementation of the system were not forthcoming at the time of roll-out [3]. This resulted in the development of needs-based, setting-dependent paper- or electronic M&E systems by various government and non-government stakeholders to report programme outcomes for different objectives. These vertical M&E systems ran parallel to the District Health Information System used to monitor overall health performance at district level. Kawonga and colleagues discuss the limitations inherent to such a vertical system in terms of its efficiency and utility in addition to the constraints on access to HIV data for programme planning at district level [4].

**Fig 1:**
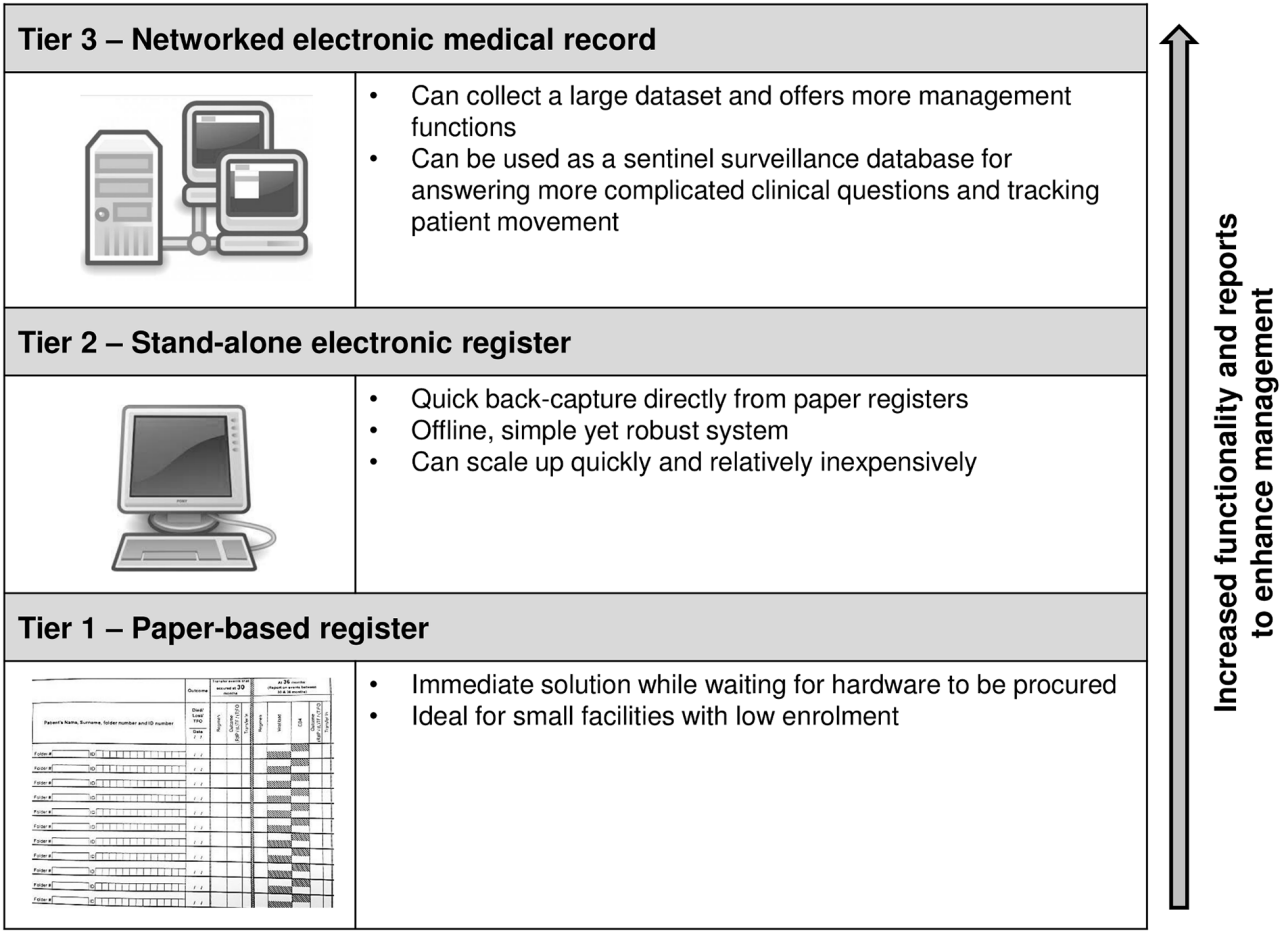
The South African national strategy to standardise M&E of patients on ART. The three-tiered approach comprises paper-based, stand-alone electronic, and networked electronic medical records systems for monitoring and evaluation of the South African ART programme.

A pragmatic approach to ART programme M&E was developed in the Western Cape Province of South Africa and is explained in details by Osler *et al*. [5]. This three-tiered approach comprises paper-based, stand-alone electronic, and networked electronic medical records systems for the ART programme (Fig 1). Each tier was developed by a different stakeholder in ART M&E across the province. The electronic register (tier 2) allows simple digitisation of the paper-based registers (tier 1) and functions as a bridging solution between paper-based registers and an eventual networked electronic medical records system (tier 3). The electronic register was developed by the Centre for Infectious Disease Epidemiology and Research (CIDER) at the University of Cape Town [6]. All three systems capture the same minimum essential monthly and quarterly ART data sets required by the National Department of Health. Supporting the three-tier framework is standardised ART clinical stationery which is included in every HIV-positive patient’s folder and which prompts clinicians for required information.

In December 2010 the three-tier ART M&E strategy was adopted by the National Department of Health to monitor ART programme outcomes in all provinces [5]. This strategy standardises the collection of key ART indicators and is supported by the District Health Information System [5]. The limitations of the paper-based register for monitoring the rapidly expanding ART programme have meant that implementation of the electronic register was prioritised in the national strategy.

No formal assessment of the implementation of the electronic register has been completed. This paper engages with theory on implementation to identify and contextualise enabling and constraining factors for implementation of the electronic register, to describe experiences and use of the register, and to make recommendations for its implementation in similar settings where standardisation of ART M&E has not been achieved.

Theory on implementation highlights important barriers to implementation that may arise at various levels of the health system, affecting the fidelity of the implementation process as well as the overall outcomes of implementation. Evaluation of both the implementation process (formative outcomes) and endpoint or summative outcomes of implementation can “optimise intervention benefits, prolong sustainability of the intervention in that context, and promote dissemination of findings into other contexts” [7]. One such theory we consider in this paper is the Consolidated Framework for Implementation Research (CFIR) [7].

### The 12 step implementation process for the electronic register

**Fig 2:**
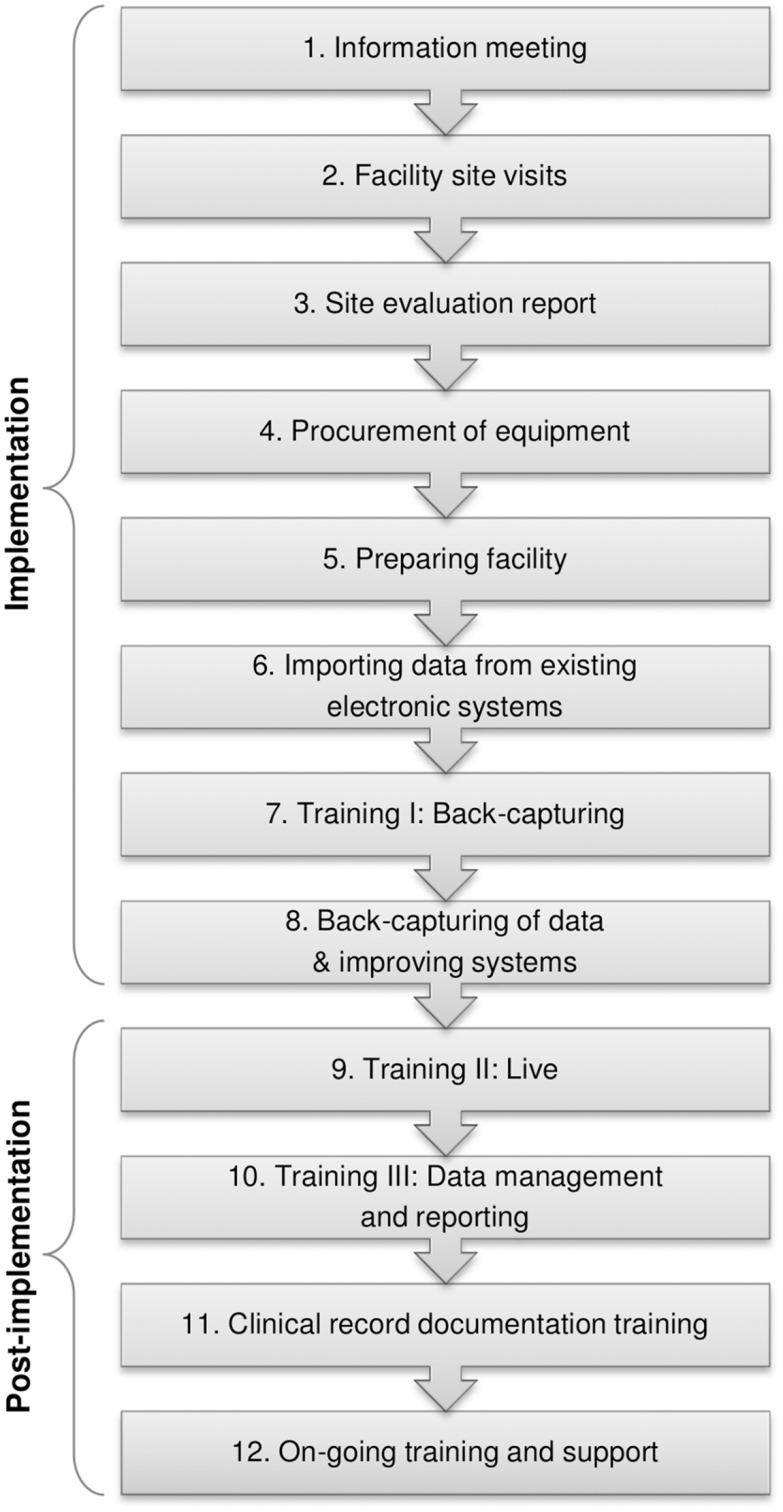
The 12-step implementation process for the electronic register. A 12-step implementation process for the electronic register was collaboratively developed by CIDER and the Anova Health Institute. Master training based on this 12-step implementation process aimed to structure and standardise implementation practices for the electronic register among the key implementers across all South African districts.

A 12-step implementation process for the electronic register was collaboratively developed by CIDER and the Anova Health Institute (Fig 2). Master training based on this 12-step implementation process aimed to structure and standardise implementation practices among the key implementers across all South African districts. This process is explained in detail:

The first step involves stakeholder meetings aimed at ART Programme Coordinators, primary health care (PHC) managers, pharmacists, as well as facility managers, ART nurses, doctors, and administrative clerks. During these meetings, the electronic register is introduced to staff members, its use along with that of the clinical stationery are discussed, whilst the requirements for roll-out including the timeline, equipment, staffing, and training needs are planned in detail. Facility visits are conducted to identify available resources such as computers, space in the facility, the number of ART patients remaining in care, the current data flow, day-to-day operational functions, previous record systems, and staffing. A facility evaluation report outlining the situation at each facility is developed to map out the recommended implementation.

Facilities are readied for roll-out, equipment (computer, memory stick, access to printer) is procured, and administrative clerks are deployed if required. M&E logistics are evaluated and adjusted at facility-level. All individuals involved are trained on use of the electronic register, general HIV and ART information, and their roles and responsibilities in implementing, reporting, and maintaining the register. Thereafter, current data from the paper-based register are captured onto the electronic register. That is, every patient who received ART at the facility, including those who defaulted on their ART, those who have died, and those who transferred-out to another facility, are captured. Once this process of back-capturing is completed, data clean-up is pursued. This important phase of the process requires administrative clerks to export data to Microsoft Excel and to perform specific checks to identify outliers and spurious reports in the data.

The subsequent phase of the implementation process, referred to as “post-implementation,” necessitates the “live” capturing of patient data directly from clinical stationery instead of the paper-based register. In routine practice, clinicians ensure that clinical stationery is completed and that patient folders are returned to the administrative clerk for data capturing prior to being filed. Administrative clerks are responsible for capturing data into the electronic register and for generating routine facility reports. These reports are signed off by facility managers, who maintain overall responsibility for programme data. Facility reports are sent to sub-district and district information management who ensure that data are captured on the District Health Information System. These individuals provide feedback on data quality to facilities. Facilities receive on-going training and support, including updates of newer software versions as well as technical support.

## Methods

The Cape Winelands district of the Western Cape, South Africa, is a rural region with a population of approximately 750000 people and comprises five sub-districts, namely Breede Valley, Drakenstein, Langeberg, Stellenbosch, and Witzenberg. More than 12000 patients were recorded in the electronic register as receiving ART through public health facilities in the district in June 2012 (Personal communication, TH).

Roll-out of the electronic register in the Cape Winelands district was initiated in public health care facilities in 2010. At the time, facilities providing ART were using paper-based registers to record ART M&E as was standard practice with the initiation of services at each new site; a standard ART M&E policy had not yet been developed. Between 2008 and 2012 the number of facilities providing ART services in the Cape Winelands had more than doubled, and the number of patients accessing ART showed a similar trend, increasing from roughly 2000 patients on treatment in 2007, to more than 4500 on treatment in 2012. The digitisation of data from paper-based registers came at an opportune time, as the paper registers were manually maintained and could become cumbersome to report on at monthly and quarterly intervals, at older sites especially. The District Implementation Teams in the Cape Winelands asked their development partner, the Anova Health Institute (from here on referred to as the partner implementer), to assist with roll-out of the electronic register.

Roll-out began with a centralised accredited ART facility in each sub-district and the programme was subsequently scaled-up throughout the district. In this paper we refer to centralised accredited sites such as hospitals or Community Day Centres that first delivered ART, as “mother” facilities, and those facilities that later accepted the expansion of ART through integration of existing patients and enrolment of new patients, as decentralised facilities.

A diverse sample of stakeholders was recruited at facility, sub-district, and district level via telephone or e-mail. The facility-level participants were selected across the five sub-districts; one facility per sub-district was included in the sample. The selection of facilities considered the following: to compile a diverse sample determined by the number of patients on ART, decentralisation status, and the historic quality of their ART paper-based register (Table 1). Data were collected from January to June 2012 by in-depth semi-structured interviews. Interviewees were invited to participate based on their involvement in the ART programme and experience with the implementation of the electronic register.

**Table 1:** Characteristics of facilities selected.

Facility	ART service available (years)	Approx. no. of patients on ART	Decentralisation status	No. of clerks during back-capture	Number of patient records back-captured	Staff dedicated to ART programme	Perceived quality of Paper Register*	Data champion present	Time to complete back-capture onto electronic register
A	>5	~1300 (large)	“Mother” facility	2	2624	Yes	Low	No	3 months
B	<5	~600 (med)	Decentralised facility	N/A	N/A	Yes	N/A	Yes	“Live” capturing
C	>5	~400 (small)	Decentralised facility	1	577	Yes	Med	Yes	1 month
D	<5	~300 (small)	Decentralised facility	2	435	No	Low	No	5–6 months
E	>5	~1500 (large)	“Mother” facility	2	2343	Yes	High	Yes	4–5 months

Table 1 shows the five facilities selected for inclusion in our sample. Selection considered the following: to compile a diverse sample determined by the number of patients on ART, decentralisation status, and the historic quality of their ART paper-based register.

* Paper register quality was determined during site visits.

Twenty in-depth interviews were conducted with staff at various levels of the health system in their language of choice. Facility-level participants were administrative clerks, ART nurses, and facility managers. At district and sub-district levels respondents were persons involved in information management. Altogether 8 administrative clerks, 4 ART nurses, 3 facility managers, and 5 sub-district/district respondents were interviewed. In two instances the ART nurse was also the operational manager of ART services at the facility. Separate interview guides were developed by the research team for use with facility-based and (sub)district respondents. The guides consisted of open-ended questions with set probes, as well as Likert-scale questions to ease comparison between responses on specific questions relating to implementation. Questions focused on respondents’ experiences of the implementation process and the perceived impact of the electronic register on the quality, access, and use of ART data as well as on service delivery.

Interviews were transcribed and where necessary, translated from Afrikaans to English by the researchers. Responses were coded by a team of three researchers (MvH, NF, and HM) and analysed using a thematic approach. The Likert-scaled answers provided an overview of responses per respondent-type and facility, and were expected to highlight inconsistencies. Two individuals (TH and CJG) affiliated with the partner implementer facilitated the roll-out process and reviewed the preliminary findings of the coding team, assisting with data interpretation and contextualisation.

After initial analysis an implementation science framework, the CFIR, provided a theoretical basis through which to further interpret the findings and allowed it to be described in consistent terminology or *constructs* that are shared across implementation theories [7]. The CFIR constructs relevant to our study are presented in Table 2 and their significance is considered in more detail in the discussion section of this paper.

**Table 2:** CFIR constructs and definitions.

Construct	Definition
**Domain 1: Intervention characteristics**	
*Complexity*	Perceived difficulty of implementation, reflected by duration, scope, radicalness, disruptiveness, centrality, intricacy and number of steps required to implement.
*Relative advantage*	Perception of the advantage of implementing the intervention vs. an alternative solution.
**Domain 2: Outer setting**	
*External Policy & Incentives*	Includes external strategies to spread interventions including policy and regulations, external mandates, recommendations and guidelines.
**Domain 3: Inner setting**	
*Implementation climate*	The absorptive capacity for change, shared receptivity of involved individuals to an intervention and the extent to which use of that intervention will be rewarded, supported, and expected within their organisation. Implementation climate comprises constructs such as *tension for change* and *compatibility*.
*Readiness for implementation*	Tangible and immediate indicators of organisational commitment to its decision to implement an intervention. *Readiness for implementation* comprises constructs such as *available resources* and *access to knowledge and information*.
**Domain 4: Individual characteristics**	
*Self-efficacy*	Individual belief in their own capabilities to execute courses of action to achieve implementation goals.
**Domain 5: Process**	
*Engaging*	Attracting and involving appropriate individuals in the implementation and use of the intervention through a combined strategy of social marketing, education, role modelling, training, and other similar activities. *Engaging* comprises constructs such as *champions* and *external change agents*.

Table 2 shows the CFIR constructs relevant to our study. The five domains of the CFIR apply to the following specific aspects of implementation of the electronic register. Firstly, *intervention characteristics* refer to the features of the electronic register. Secondly, *outer setting* refers to the organisations and the relationships between organisations that influence implementation. In this case these include the provincial and district Departments of Health, the developers of the electronic register (CIDER), and the partner implementer (Anova). Thirdly, the *inner setting* includes the facility as well as the staff, which overlaps with the fourth, *individual characteristics* of those involved in implementation. Lastly, the *process* refers to the activities that comprise the implementation of the electronic register.

In this study the five domains of the CFIR, namely *intervention characteristics*, *outer setting*, *inner setting*, *characteristics of individuals*, and *process*, apply to the following specific aspects of implementation of the electronic register. Firstly, *intervention characteristics* refer to the features of the electronic register. Secondly, *outer setting* refers to the organisations and the relationships between organisations that influence implementation. In this case these include the provincial and district Departments of Health, the developers of the electronic register (CIDER), and the partner implementer (Anova). Thirdly, the *inner setting* includes the facility as well as the staff, which overlaps with the fourth, *individual characteristics* of those involved in implementation. Lastly, the *process* refers to the activities that comprise the implementation of the electronic register.

### Ethics Statement

All study participants gave written consent to participate in a personal audio-recorded interview. The protocol and consent procedures were approved by the Health Research Ethics Committee, Faculty of Health Sciences, University of Cape Town and the Western Cape Provincial Research Health Committee (both in South Africa).

## Results

We identified seven themes with ten characteristics that describe implementation of the electronic register in the Cape Winelands district. These characteristics fall within the domains and constructs of the CFIR which serves to illustrate how they enable or constrain implementation (Tables 3 and 4), and are elaborated upon in the discussion section.

**Table 3:** Summary of key findings.

Themes	Key quotes
**Ease of implementation of the electronic register**	*The programme itself is not difficult*, *because we work on ETR*.*Net*, *[the electronic TB register]*, *and it is exactly the same*.
**The perceived value of an electronic M&E system**	*I was willing [to have the system implemented] because I know electronic things work faster and are more accurate [than paper]*.
**Importance of stakeholder engagement**	*You have to do a bottoms-up approach […] then you go wider*.
**Influence of a data champion**	*I think [the administrative clerk] took to leadership […] When he was here [the electronic register] was implemented quite well […] He was exposed to [the programme]; he had the experience*.
**Operational and logistical factors**	*We were capturing information that we were not sure*, *is this correct*? *[…] And then it was a lot of work because we had to [capture] all the patients from 2004*.
	*It is not the system*, *[the electronic register]*, *itself [that is difficult]*. *It is the other practical things [involved in integrating ART services]*.
**Workload and role clarity**	*[Back-capturing] had taken months and months [to be completed…] That is perhaps where it needs clarity […] Who takes responsibility for the stats*?
**Importance of integrating the electronic register with routine facility M&E**	*[The PHC administrative clerks] capture all the other [facility] data*, *but [the electronic register] is almost a mystical something which is apart as ARVs are*.

Table 3 is a summary of the results expressed in seven themes with illustrative quotes.

**Table 4:** Overview of the CFIR domains, salient constructs, and complimentary characteristics.

CFIR Construct	Corresponding characteristic(s)
**Domain 1: Intervention characteristics**	
*Relative advantage*	1. Buy-in from management and facility-level staff
*Complexity*	2. Quality of paper registers
	3. Workload
**Domain 2: Outer setting**	
*External Policy & Incentives*	4. Leadership from provincial Department of Health
**Domain 3: Inner setting**	
*Implementation climate*	5. Level of ART service integration / maturity of ART services
*Readiness for implementation*	6. Resources (material, human, physical space, training)
	7. Role clarity
**Domain 4: Individual characteristics**	
*Self-efficacy*	8. Experience with ART programme M&E, level of computer literacy, and personal self-efficacy
**Domain 5: Process**	
*Engaging*	9. Champion
	10. Facilitation and support from partner implementer

Table 4 shows ten characteristics that fall within the domains and constructs of the CFIR to describe enabling and constraining factors for implementation.

### Ease of implementation of the electronic register

Respondents were asked to indicate their agreement with the statement, “the process of implementation of the electronic register was very difficult” on a scale of 1 to 5 (1 showing strong disagreement and 5 showing strong agreement). The majority of respondents did not find implementation of the electronic register difficult. The extent to which respondents perceived implementation to be difficult varied by respondent type and in two cases between respondents from the same facility. Reasons cited for positive experiences included: familiarity and experience with the paper register or with other electronic tools such as the TB register (ETR.Net) and a local paediatric ART register; receiving training on the electronic register; and the intensity of support from the partner implementer during facility preparation and roll-out.

The programme itself is not difficult, because we work on ETR.Net, [the electronic TB (Tuberculosis) register], and it is exactly the same.

[Respondent 17, Sub-district/District management]

I didn’t find anything difficult [about implementation]. I knew how to work with this [register] because we had the training before.

[Respondent 5, Administrative clerk, Facility B]

I would say [implementation] went relatively well here, because [the partner implementer] helped us with the roll-out and training on the electronic programme.

[Respondent 16, Sub-district/District management]

Someone who doesn’t have speed [with a computer] will naturally find [implementation] difficult.

[Respondent 13, Administrative clerk, Facility E]

### The perceived value of an electronic M&E system

All twenty respondents thought that the electronic register had advantages over a paper-based M&E system. The perceived advantages of an electronic register positively influenced the ease with which the system was adopted for implementation.

I was willing [to have the register implemented] because I know electronic things work faster and are more accurate [than paper].

[Respondent 15, ART nurse/Facility manager, Facility E]

There were not really many hiccups [in terms of achieving buy-in] since [the electronic register] was introduced for roll-out or any resistance from management that I can think of. We realised the programme was going to help us.

[Respondent 16, Sub-district/District management]

All respondents scored positive responses on a Likert-scale when asked whether the implementation of the electronic register had a positive impact on their work. In reflecting on their experiences of using the electronic register post-implementation, perceived advantages of the register varied by respondent type and demonstrated how the system held distinct benefits for each work role. Administrative clerks enjoyed being able to produce faster and more accurate monthly and quarterly facility reports:

I struggled with [aggregation of] the quarterly report [from the paper register] because I had to count everything. [The electronic register] produces the list automatically. It is just a click of the button.

[Respondent 2, Administrative clerk, Facility A]

Nurses explained that they had increased access to patient data for day-to-day patient-specific queries (e.g. blood results, last facility visit date, drug regimen). Facility managers noted that their knowledge of the HIV-positive cohort attending their facility has improved, while sub-district and district respondents specified that they had immediate access to facility-based data.

I update the dispatches myself. [The electronic register] has made it much easier to zoom in on the ART programme, to equip and empower myself. It has made it easier for me to plan, and to give answers.

[Respondent 16, Sub-district/District management]

Positive experiences with electronic ART data were tempered by mistrust of data at facilities that had poor quality paper registers as the source data, administrative clerks who were new to the ART programme, or where the regularity of data capturing post-implementation was a concern. Such concerns were only expressed by facility managers and sub-district respondents, who in some cases were uncertain of administrative clerks’ ability to capture medical information accurately, or were anxious regarding the maintenance of ART data post-implementation.

The system is good if [the data] are just entered correctly. That is one of the gaps, because if it is not entered correctly, then I cannot get the correct information […] We still have the medical background. I know about CD4s and those things. Those children [i.e., the administrative clerks] sometimes know nothing about these things.

[Respondent 12, Facility manager, Facility D]

### Importance of stakeholder engagement

The buy-in meeting was perceived to be the gateway to implementation. Sub-district and district respondents noted that drawing in key role players at facility-level, i.e., the facility manager, ART nurses, and administrative clerks, would enable the implementation process.

[Buy-in] is very important. In our sub-district we immediately pulled in our Senior Administrative Officer, all the administrative clerks in the clinics, they were part of the meeting when [the register was introduced for roll-out]. You have to do a bottoms-up approach, first do it in a sub-district, let people see that it works and then you go wider. Let a small sub-district show you it can work and win over the [district] Director.

[Respondent 18, Sub-district/District management]

ART Programme Coordinators generally do not have a line management function over the clinics’ administrative clerks and nursing staff. [They] are here only to provide support to the programme, because your operational managers are the line managers. So [facility-level staff] must be involved from the start and be part of the story [in order for implementation to be successful].

[Respondent 16, Sub-district/District management]

Buy-in for implementation was exhibited in various ways by facility-level staff, such as through the readiness with which a facility manager freed her staff for training, consideration for the administrative clerk during the back-capturing phase, eagerness with which space was made available to accommodate the system’s requirements, openness of staff to receive training, or watchfulness over the patient folder flow so that data could be captured timeously.

In addition to overall willingness, support by the partner implementer was consistently emphasised by all respondent types as facilitating implementation in terms of tangible and intangible system requirements. This included:

- Provision of resources such as computers and administrative clerks during the back-capturing step;
- Training and mentoring on the electronic register and correct use of clinical stationery; and
- Training administrative clerks on basic HIV and ART knowledge to facilitate data capturing.

Support from the partner implementer enabled role players to overcome potential obstacles to implementation. The on-going support provided by the partner implementer was important to encourage respondents to overcome challenges during implementation and to ensure that implementation would continue.

The thing that made [implementation] the easiest for me was [the partner implementer], his expertise about [the electronic register], he was hands-on. [The partner implementer] trained everybody [on the electronic register]. It was not like train-the-trainer; the trainer was there. If I just tried to tell him that something is not going to work then he told me, “Come on!” and he makes it work. It was one of the nicest projects [I did] in the [sub-district].

[Respondent 18, Sub-district/District management]

### Influence of a data champion

Administrative clerks’ experience with ART programme M&E, level of computer literacy, and personal self-efficacy were found to develop the confidence of others in the facility for implementation. Where such highly-motivated individuals or *champions* were present during implementation, respondents communicated positive experiences of implementation.

I think [the administrative clerk] took to leadership. He and I sat together for an hour, just so that he could show me [on the register]. Then I realised that [the electronic register] is similar to the ETR.Net (the TB register). When he was here [the electronic register] was implemented quite well. It is because of the training that he had already received. He was exposed to [the ART programme]; he had the experience.

[Respondent 7, Facility manager, Facility B]

Such positive experiences during implementation carried over into the post-implementation context as clinical staff trusted the data champion to take responsibility for the maintenance of the programme.

I think [implementation] has been successful because the administrative clerk has been trained very well on how to [capture data]. And she has made a point to do it on a daily basis and in an exact way, so the [data] has been up to date up until now.

[Respondent 9, ART nurse, Facility C]

Conversely, respondents at facilities without a data champion—either during implementation or post-implementation—exhibited fewer positive experiences of implementation and showed uncertainty over the maintenance of the data for the ART programme.

[Our current administrative clerk] used to be an administrative clerk at [another clinic]. She was taught to pull folders at [reception] without a computer. She didn’t have the experience. See, she wasn’t exposed to ARV (antiretroviral) information. She learnt on [the electronic register] here [at the clinic]. They are busy training her now, and eventually there will be an improvement [in how the data is managed].

[Respondent 7, Facility manager, Facility B]

### Operational and logistical factors

Several operational and logistical factors affected implementation. Respondents singled out the back-capturing process as the most challenging factor; other important factors were availability of resources required for implementation, and the level of integration of ART services in the facility.

#### Back-capturing

The back-capturing process was the most time-consuming step of the implementation process, ranging between one and five months to complete for the five facilities. The number of patients historically on ART (which related to the back-capturing workload) and poor quality paper registers protracted back-capturing:

We were capturing information that we were not sure, is this correct? Because it was the information we got from the paper register not from the [patient] files. And then it was a lot of work because we had to [capture] all the patients from 2004 [*emphatically*].

[Respondent 11, Administrative clerk, Facility D]

The strain of the back-capturing process was concentrated among administrative clerks as they were not exempt from fulfilling their general administrative duties during this time. The back-capturing process also had broader implications for the facility and other facility staff. A nurse explains:

[The back-capturing process] definitely had an impact on our workload on the PHC side, because [the administrative clerk] used to help pulling the files and process patients at reception, so when she [was busy back-capturing data] patients had to wait longer periods for the folders to get through.

[Respondent 9, ART nurse, Facility C]

At facilities with more than one administrative clerk, reception and back-capturing duties could be shared, causing less disruption to the facility and alleviating, but not removing, the strain on the administrative clerks in fulfilling their allotted duties.

#### Availability of Resources

In some facilities, reorganising space to accommodate the electronic register was problematic as the system requirements, i.e., a space for the computer and the administrative clerk/s, were not immediately supported by the available infrastructure.

[The administrative clerk] made a space for herself in that corner and you know we are very cramped. I get claustrophobia for her in that corner. She asked me to be separate because it is not private for the patient to sit in [the consultation room] and everyone is there. Confidentiality and my information—the entire world knows my information.

[Respondent 10, Facility manager, Facility C]

Within their capacity, the partner implementer provided additional resources such as computers, printers, and administrative clerks to assist with back-capturing where required. Additional clerks were assigned to facilities with high workloads (such as Facility A and E), or to facilities with especially challenging conditions such as Facility D (discussed in the following section). Respondents saw support in terms of resource allocations as a crucial factor that facilitated implementation at facility-level:

I know that [the partner implementer] donated many of the computers and printers. It takes long [to acquire resources] because everything has to go through the government; there is red tape. So the [partner implementer] really helped us there.

[Respondent 16, Sub-district/District management]

#### Timing of roll-out of ART services and the electronic register

Four of the five facilities had established ART services prior to implementation, which facilitated the implementation process. Staff at such facilities reported that they could more easily integrate the steps of the implementation process with their typical responsibilities surrounding ART services and data at the facility.

Look, it is basically the same as the [paediatric ART register]. You used to enter [patient] visits on it, now you just enter the visits on [the electronic register]. So it wasn’t like there is now something [new], that there was no system before.

[Respondent 8, Administrative clerk, Facility C]

Existing ART services also had an impact on the importance given to training staff on the electronic register, as well as consideration for administrative clerks during implementation. The facility manager from Facility C explains:

[The implementation of the electronic register] did not have an impact [on the facility] at all […] It is the same as any other normal trainings, so you have to make provision for that. When staff have gone for training then you must make other staff available. It was a normal process; we must ensure that all the sections are covered.

[Respondent 10, Facility manager, Facility C]

We realised that [back-capturing] is an extra load on [the administrative clerk]. That is why we loosened her so that she could go on [to back-capture]. It was important that the work must be done.

[Respondent 10, Facility manager, Facility C]

In contrast to the other facilities, Facility D experienced the simultaneous roll-out of ART services and the electronic register. All respondents from this facility shared negative experiences of the implementation process.

That is why [implementation] was difficult, because we had to integrate [ART patient folders] with our folders [at the same time as the electronic register was implemented] and we didn’t have space. It is not the system, [the electronic register], itself. It is the other practical things [involved in integrating ART services].

[Respondent 12, Facility manager, Facility D]

In the latter example, operational challenges during implementation were much more pronounced, and these were compounded by complexities such as poor quality paper registers and limited space.

### Workload and role clarity

Despite having favourable factors to suggest successful implementation, the roll-out process was hampered at Facility E due to a high workload and perceived lack of leadership. Back-capturing took four to five months to complete given a high workload and confusion regarding responsibilities of administrative clerks and the procedure for reporting ART data during this time. Respondents suggested that clearer communication regarding the above would have helped implementation.

[Back-capturing] had taken months and months [to be completed] and every time we get to meetings it is only [this facility] which is still in [back-capturing] mode. No one asked me [to help], and I just went on [with my work]. But we couldn’t send in our statistics while we were back-capturing. And that is perhaps where it needs clarity—whether the senior administrative clerk is also a data capturer. Who takes responsibility for the stats?

[Respondent 13, Administrative clerk, Facility E]

### Importance of integrating the electronic register with routine facility M&E

Respondents consistently raised the importance of integrating the electronic register with routine M&E structures. Post-implementation, respondents noted that there was an occasional operational challenge with the maintenance of ART data as administrative clerks who worked primarily with PHC data did not take accountability or ownership of ART data as routine facility data. This situation created complications for the maintenance of ART data when the designated ART administrative clerk was absent. In such instances, administrative clerks reported that there would be a backlog of ART information to be captured, or that uncaptured folders would be refiled, which could compromise data quality.

Some administrative clerks stressed their attempts to provide in-service training for their PHC counterparts who had not received training on the electronic register, so as to overcome the issue of data maintenance by other clerks. They maintained that their efforts were often met with resistance.

According to [the PHC administrative clerks], I am the administrative clerk for the ARV-clinic so I can just go on capturing the data for [the electronic register]. I don’t know if they will also [capture the data], they are not interested. I don’t know if they are scared of [the electronic register] or what.

[Respondent 8, Administrative clerk, Facility C]

Sub-district and district managers explained this situation by emphasising that the lack of ownership of ART data in the facilities was part of a larger issue dating back to the vertical roll-out of the ART programme and vertical reporting systems and data flows for ART M&E.

[The PHC administrative clerks] capture all the other [facility] data, but [the electronic register] is almost a mystical something which is apart as ARVs are. I think part of the problem is that NIMART (nurse-initiated management of ART) was not rolled-out correctly, so the ownership of the ARV patients does not lie entirely with the [PHC] clinic yet.

[Respondent 19, Sub-district/District management]

In the past ARVs were quite separate. When the call for integration came [staff] at facility-level started to realise, the operational manager, “If [ART data] comes to me to look at, to the sub-district, to the information people, to the PHC manager, then [ART patients] are our patients.” So I would think that with [the electronic register] and the integration of the ART programme, [the register] came at the right time to bring it all together.

[Respondent 16, Sub-district/District management]

Respondents pointed out that training the entire facility staff component on the electronic register would facilitate the maintenance of the data post-implementation. Administrative clerks in particular noted that drawing in various facility staff would inculcate an understanding of the importance of their role in ensuring good quality ART data, for example, through the correct completion of ART clinical stationery, facilitating folder flow, and enabling consistent data capturing.

I think in future they have to see to it that the [PHC] administrative clerks are on par with [the electronic register]. Not just to have a one-sided focus and say that it is the ARV data capturer’s problem. Because what will we do if the ARV data capturer is sick one day? Who will take over?

[Respondent 7, Facility manager, Facility B]

[The administrative clerks] have good intentions to capture all folders every day, but it doesn’t happen that way in practice. So the workload [of capturing ART data] is a big thing that was added, but there were no extra hands to do it. The administrative clerks are quite busy—you don’t see an administrative clerk standing around doing nothing […] And it is a new thing too, because they are not computer literate and I think for them it is a bit of a stressor. It is just that one needs more training sessions, follow-up training sessions.

[Respondent 19, Sub-district/District management]

## Discussion

Operationally, respondents all shared positive experiences of electronic ART M&E data. They report increased access to, and faster and more accurate reporting for ART staff at facility level as well as an increased sense of control over the ART programme for sub-district and district management. Electronic ART data are thus not only more accessible at facility-level for day-to-day patient queries, enhanced facility management, and improved reporting, data exports allow access for those who are cut-off from facility-based data, facilitating programme management and planning.

These summative endpoint outcomes are one aspect of understanding implementation. In the introduction to this paper we explained that implementation is more than *what* has been achieved: if we explore *how* and *where* implementation was achieved, we can maximise positive outcomes, increase the chances that these will be maintained in a particular setting, and are able to make recommendations to improve the likelihood that positive outcomes will be achieved in other contexts [7].

In investigating the formative rather than the summative outcomes of implementation, this discussion focuses on the most salient constructs in each of the five CFIR domains, our ten characteristics’ relation to them, as well as the interactions across domains. Such mapping of the domains and constructs and their dynamic relationships allows us to construct the scaffolding for implementation [8] drawing on the five diverse facility-settings included in our sample.

**Fig 3:**
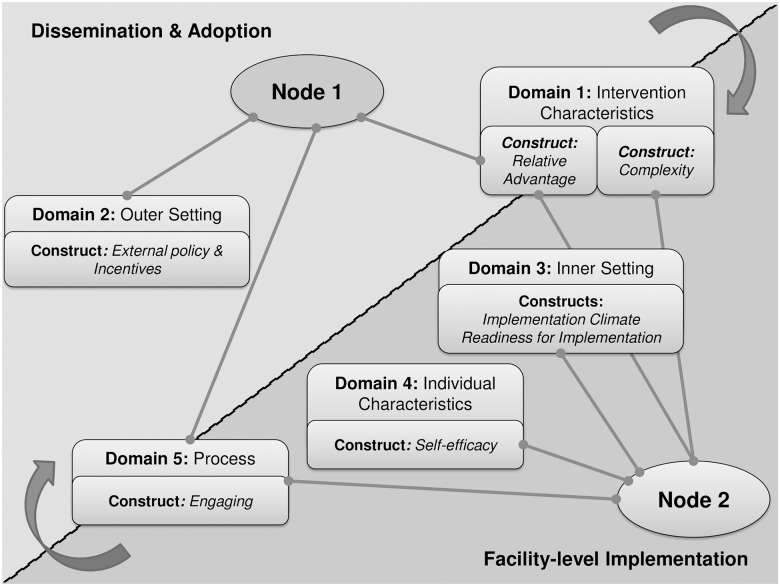
Interactions between CFIR domains and constructs. Implementation occurred at two nodes. The term “node” is used to describe the crucial intersection of CFIR domains to describe how implementation happens. Node 1 predicts the adoption of the electronic register for roll-out while Node 2 describes implementation at facility-level. Although all five domains come into play at each node, the figure shows the most important domains and constructs highlighted in our findings.

We found that implementation of the electronic register was not a linear process but that it instead occurred at two *nodes*. The term “node” is used to describe the crucial intersection of CFIR domains, the dynamics of which illustrate how implementation occurs. The first node describes the adoption of the electronic register for implementation and the second node involves implementation at facility-level. Although all five domains are significant at each node, the discussion focuses on the most salient domains and constructs to contextualise two stages of implementation, as related in our findings. These nodes and their interactions are illustrated in Fig 3.

### Node 1: Adoption of a standardised electronic ART M&E register

The first node covers the dissemination and adoption of the electronic register for implementation: the *intervention characteristics*, *outer setting*, and *process* are the most appropriate domains to describe this part of implementation. *External policy & incentives*, or leadership from provincial Department of Health, is related to the outer setting and was an important catalyst for implementation as it mobilised key stakeholders in ART M&E to vie for roll-out of the tool in districts in the Western Cape. Such endorsement of the electronic register, what Greenhalgh *et al*. [9] term “political ‘must-dos,’” facilitated buy-in for the intervention.

A key dynamic in the dissemination of the electronic register and a precursor to its implementation at facility-level was the *relative advantage* of the register as well as *engagement* (in this case from the partner implementer). This is in line with Greenhalgh *et al*. [9] who maintain that relative advantage is a requirement for adoption or *buy-in* from stakeholders. The authors explain that despite the relative advantage or strong evidence in support of an intervention, buy-in is not automatic but requires a process of engagement to clarify the relative advantage of the intervention to potential recipients. This involves dissemination of information regarding the intervention, development of an implementation plan, and support of those involved in implementation in carrying out the plan.

Our findings suggest that as a simple electronic bridging solution to the paper-based register, the relative advantage of the electronic register was immediately apparent to district and sub-district stakeholders and facility-level staff. The electronic register offered simple digitisation of ART data, making it compact, allowing health care workers “click-of-a-button” access to patient-level data, faster and easier reporting, and increased accessibility of data for facility, sub-district, and district management, while requiring comparatively few resources. The CFIR refers to individuals who “formally influence or facilitate intervention decisions in a desirable direction” as *external change agents* [7]. In this instance, buy-in was driven by a partner implementer at sub-district and district level. The longstanding relationship between the partner implementer and the Department of Health in the Cape Winelands arguably also helped the buy-in process, as did the similarity in backgrounds of those involved in implementation, as suggested by Greenhalgh *et al*. [9]. Although such a driver took the form of an outside organisation, we suggest that engagement need not come from an external organisation; an internal motivated individual or individuals could achieve the same dynamism.

In summary, the relative advantage of an intervention paired with engagement from enthusiastic individual(s) who take responsibility to drive implementation are critical for successful adoption of an intervention. In this case, policy & incentives played a role in setting the agenda for roll-out yet it cannot substitute the power of an intervention that makes sense to those involved, and key individuals who help to sell the intervention to those potentially involved in implementation.

### Node 2: Implementation at facility-level—a complexity versus compatibility balance

The second node refers to implementation at facility-level. Most of the ten characteristics we found influenced implementation of the electronic register intersect at this node which hints at the complicated nature of implementation at ground-level. The key domains are *intervention characteristics*, *inner setting*, *individuals involved*, and *process*. Interaction between these domains is multifaceted; as Damschroder *et al*. [7] suggest, “without adaptation, interventions usually come to a setting as a poor fit, resisted by individuals who will be affected by the intervention, and requiring an active process to engage individuals in order to accomplish implementation.” This statement encapsulates the four domains highlighted by our findings. At ground-level each of these components of implementation are at their most complex. Abbott *et al*. [8] describe health care facilities as “complex and adaptive systems” where “methods that work in one organisation or location may fail in another,” emphasising that implementation is context-dependent. Moreover, Greenhalgh *et al*. [9] maintain that implementation is social in so far as it involves individuals. The authors explain that

People are not passive recipients of innovations […] They seek innovations, experiment with them, evaluate them, find (or fail to find) meaning in them, develop feelings (positive or negative) about them, challenge them, worry about them, complain about them, “work around” them, gain experience with them, modify them to fit particular tasks, and try to improve or redesign them—often through dialogue with other users.

Intervention characteristics—*relative advantage* and *complexity*—were particularly important for facility-level implementation as they influenced the receptivity of the inner setting for implementation. *Relative advantage* facilitated buy-in of facility-level staff, especially in large mature facilities where paper-based registers were becoming more and more cumbersome. Under such circumstances there was an added *tension for change* (as opposed to younger or ART-naïve facilities). We found that *complexity* of the intervention, which comprises amongst others the perceived difficulty, duration, and number of steps required to implement, was not specific to the intervention. Instead, *complexity* was influenced by factors in the inner setting and characteristics of individuals involved in implementation. Osler *et al*.[5] explain that

with each higher tier comes added complexity and the need for additional support. The second-tier solutions [such as the electronic register] require computer literate staff, computer availability, and software and hardware support, in addition to the training and protected staff time that are common requirements of all tiers.

Key inner setting factors included the quality of paper registers, workload (which related to the maturity of ART services), resources (material, human, physical space, training), level of ART service integration, as well as experience with ART programme M&E, level of computer literacy, and personal self-efficacy of individuals involved. These factors informed the *implementation climate* (in terms of the intervention’s *compatibility* with existing workflows and systems), the *readiness for implementation* (in terms of *available resources* and *access to knowledge and information*) of facilities, and the *self-efficacy* of individuals involved to achieve successful implementation. Implementation was notably easier in facilities which rated favourably in these characteristics and constructs.

Even in facilities where most system requirements were in place prior to implementation, interaction between the intervention, inner setting, and individuals involved were not necessarily favourable. As with node 1 implementation required an active process of engagement. At facility-level, this relied on two actors namely the data *champion* and *external change agent*. These key role-players facilitated the complexity of the intervention, compatibility and readiness for implementation in the inner setting, and self-efficacy of individuals involved.

Data *champions* positively influenced implementation by making themselves accountable for the success of implementation, holding involved others accountable, and developing the confidence of less-involved others by drawing on their personal self-efficacy. Data champions by definition exhibited pride in their work and an enthusiasm to learn, which could overcome initial shortcomings in skill (computer literacy and experience with the ART programme M&E for instance); the latter capacities could be developed through training and support. In terms of optimal implementation, data champions are a necessity as they are able to arbitrate and potentially overcome barriers to implementation as they arise at ground-level.

Whereas data champions were important for operations at facility-level, the *external change agent* played a critical role in achieving buy-in of facility-level staff, facilitating the implementation climate (compatibility), readiness for implementation (in terms of available resources and access to knowledge & information), and developing the self-efficacy of individuals involved. As evidenced in our interviews, such an individual who drives implementation at various levels—district, sub-district, or facility—is invaluable in maintaining the momentum for implementation and responding to needs as they arise, be they related to individual skills development or infrastructural requirements for implementation.

In summary, implementation at facility-level is complex as it entertains various components of implementation which are of themselves complex: the intervention, inner setting, and individuals involved. To achieve successful implementation at facility-level, the interaction between these components requires supervision and facilitation by individual drivers of implementation. In our five facility contexts, data champions and an external change agent took on this role in their varying capacities to overcome challenges and barriers to implementation. Yet, despite less desirable factors and the absence of a data champion in some facilities, our findings show that successful implementation (in terms of summative outcomes, i.e., regular reporting) can still be achieved.

Based on the preceding discussion of implementation at the two nodes, i.e., at the adoption of the electronic register for roll-out, and at implementation at facility-level, we found that r*elative advantage*, an intervention characteristic construct, and *engagement*, a process construct, were the only constructs to overlap between nodes. This suggests that these constructs are critical aspects of implementation which have the capacity to influence outcomes of implementation at both adoption and coalface stages of implementation.

### Strengths and limitations of the study

We identified a number of strengths and limitations in our work. The primary strength was the qualitative nature of the methodology. The nuance and complexity of implementing any new intervention, at least in this setting, was best measured through in-depth interviews. For the scaled-answer questions a respondent may indicate one thing, e.g. that the implementation process was not difficult, while during in-depth interviews various challenges would be revealed. Also as Abbott *et al*. [8] identify, implementation science is in its infancy and qualitative approaches are ideal to collect the various constructs and casual pathways for which quantitative approaches can be used in future. One of Abbott *et al*.’s [8] identified best practices include to “understand how the multiple levels of complex interventions intersect and how they relate to the intervention. We were well-aligned to this as we interviewed with multiple levels of the District Health System (facility, sub-district, and district level) as well as interviewing across cadres within the facility (administrative clerk, nurse, and facility manager).

Limitations of the research include limited generalisability across South Africa, methodological limitations relating to the facilities and respondents included in our study sample, and potential researcher and respondent bias.

South Africa is a diverse and complex nation, and this extends to its public sector health facilities. As such, our findings may not uniformly apply to South Africa’s fifty two districts. Findings and recommendations should be considered and tailored to each district’s context, especially as the Cape Winelands district had dedicated administrative clerks for the ART programme in each facility at the time of implementation; this is not the case everywhere. Yet, we consider our sample of facilities and respondents to sufficiently represent implementation in this context. The facilities selected include diverse facility characteristics, and we reached saturation in our interviews. It is reassuring to have found that the characteristics and constructs highlighted as important to implementation, whether positively or negatively applied, are shared across the five facilities. This gives weight to our ultimate findings on factors which influence implementation.

In terms of researcher bias, three of the interviewers and analysts were loosely affiliated with the partner implementer which may have affected their pure objectivity. To clarify, two researchers were permanently employed by Anova Health Institute, another was appointed on a contractual basis to work on this project specifically, and the last person was affiliated with a local university and not with the organisation. It is noteworthy that the partner implementer would not gain from a biased representation of implementation. Instead, the partner implementer and those interviewers and analysts loosely associated, had a vested interest in unpacking and identifying gaps in implementation in this context so as to inform and guide future implementation efforts. This is also the reason for engaging theory on implementation more broadly. This goal was a driving force for the research, and the interviewers and analysts took great care to remain objective in analysis and interpretation through systematic coding of themes and regular discussion on the interpretation of findings through an implementation framework.

Another methodological matter is whether respondents may have provided desirable answers since some of the researchers were allied with the Anova Health Institute. This is a consideration in all research but one of particular pertinence in this study given the longstanding relationship between the partner implementer which headed the evaluation and the Department of Health in the Cape Winelands. Such immersion and familiarity with a study context has both pros and cons. In this case, we believe that there were some benefits to the familiarity in terms of trust and a deeper understanding of the local cultural competency. Our findings reflect nuanced views of implementation across the five facility-contexts, including both positive and negative experiences of implementation. Although desirable answers could have been given in some instances, these were arguably overshadowed by the varying perspectives we gathered from interviewees across different levels of the health system in the district. Another consideration is that the study was conducted a year after roll-out, requiring respondents to reflect on the implementation process some time after it had been completed, i.e., while working in a post-implementation context. This could introduce recall bias. Lastly, there was a data audit component of this research, but as it only established a baseline of data quality in the electronic register, those data could not be triangulated to understand the impact of implementation on data quality. Although beyond the scope of this paper, we acknowledge that data quality is critical to successful implementation as these data inform evidence-based decision-making in the ART programme. Kaposhi *et al*. [10] outline this crucial aspect of ART M&E using the Eastern Cape province of South Africa as case study. To mitigate the issue of data quality, the Western Cape Department of Health introduced an ART M&E standard operating procedure in November 2013. Future evaluations of the electronic register should investigate the issue of data quality, implementation of the standard operating procedure, and make recommendations for the maintenance and improvement of data.

Our findings suggest a more broad evaluation of implementation characteristics that could provide a greater understanding of which domains, constructs, and interactions result in more rapid and lasting implementation of new interventions.

## Conclusion

Our findings suggest that the electronic register, tier 2 of the three-tiered framework, is an innovative improvement on the paper-based registers previously used to record M&E for the ART programme in South Africa. It is also a viable option for implementation in resource-constrained settings.

We identified seven themes and ten characteristics which highlight enabling and constraining factors for implementation of the electronic register in the Cape Winelands district. These findings were interpreted using an implementation theory—the CFIR—to ground our description of *how* implementation took place (formative outcomes) and in so doing construct the scaffolding for implementation across the five diverse facility-settings included in our sample [8]. We found that implementation occurred at two nodes, i.e., at the adoption of the electronic register for roll-out, and at implementation at facility-level. *Relative advantage*, an intervention characteristic construct, and *engagement*, a process construct, were the only constructs that were significant at both nodes. We suggest that without these critical aspects of implementation, formative and summative outcomes of implementation at both the adoption and coalface stages of implementation would be negatively affected.

Applying an implementation theory allowed our findings to be described in consistent terminology or *constructs* that are shared across implementation theories, and to highlight important considerations for roll-out of an electronic register in this setting. This approach has value in that findings from one study may inform implementation across multiple contexts and interventions. Follow-up research is expected to further understand implementation and the long-term success of implementation across the district and the country.

## Supporting Information

S1 Information Transcript excerpts from an interview with a nurse. (PDF)

S2 Information Transcript excerpts from an interview with a facility manager. (PDF)

S3 Information Transcript excerpts from an interview with a sub-district/district respondent. (PDF)

S4 Information Interview guide—Data clerks, Facility managers, and Nurses. (PDF)

S5 Information Interview guide—Sub-district/district respondents. (PDF)
