# Supplementary material for: Implementation of an Electronic Monitoring and Evaluation System for the Antiretroviral Treatment Programme in the Cape Winelands District, South Africa: A Qualitative Evaluation
Source: PLoS One. 2015 May 12;10(5):e0127223. doi: 10.1371/journal.pone.0127223 (PMC4429075; doi:10.1371/journal.pone.0127223)
Supplement: S1 Information — (PDF) [file pone.0127223.s001.pdf]

Date conducted: 03-02-2012 **Interviewee type: N**
Date transcribed: 13-02-2012 **Interviewee code: N2**
Interviewer: HM **Site: Facility E**

HM: How do you experience working with TIER.Net?
I think the hand system was much more confusing. You had to go back so many pages for someone who started in 2004. You had to...his name would be in three registers, or two registers, so it could be a mess if you started in 2001, because that register is from 0 to 36 months, and then again from 36 to 72 months. It would go into a third register if it was for a longer time period. Understand? The continuation of the...but on TIER.Net if you go onto a person's name then you can see everything, whereas you would have had to go through a few registers. So the work, the volume of work in the paper register, is now compact on the computer – just a slide, just a picture, and that is better. So he can store more information than we stored in the register.

HM: What do you like and dislike about the system?
What I like about the programme is that all the clients' information is on TIER.Net so if someone phones me from the hospital, say one of our clients has been admitted to … Hospital. And then they send me a name and surname, then they say, 'Sister, I just want to know what medication he is on.' Then I can see what medication he is on, when, what was the last CD4, when was it done. So I can give out that information. Otherwise [with the paper register] I would have had to walk to reception, look for the person's folder, come back again, call the doctor, try to get hold of the doctor, and then try to give him the information.

HM: What was different about your work, your day-to-day activities, before you had TIER.Net?
The files had to be written into the register by hand. The clerk did that, but if she now went on holiday for three weeks, the nurses had to help. When she comes back, then she has a lot of work waiting for her. Because every day is a lot of work. Every visit that that person has here at the clinic must be entered, or written into the register. So you can think for yourself… us nurses who are not as confident with the register, as I said there are three different registers that you have to check for that person.

HM: Statement: The process of TIER.Net implementation was very difficult.
4 – Agree. The clerks did not fully understand the first training they received. And the more they worked with the system the better they became, but it took the clerk a very long time to do the back-capturing. Just to enter the details of one person took very long.

HM: Did you have two clerks from the beginning?
No, we started using TIER.Net in December 2010 but [D2], the clerk who had worked there since 2004, went on holiday. In January 2011 [D6] started to back-capture and [D2] started helping her with the capturing later. [D6] did capturing in the mornings on one of the computers.

HM: Did the fact that the clerks were busy back-capturing have an impact on the clinic? On your duties?
Not really. Because [D6] did the back-capturing fulltime, while [D2] would do the other administrative duties first, such as receiving patients in the morning and entering them on the RMR sheet. Later when the clinic was quiet in the afternoons, the nurses would take over the few patients who were left, and [D2] would also help with the back-capturing. It did not affect their clinical work because they were two.

HM: Would you say there was a difference in your work during back-capturing, or before you started capturing 'live'?
We felt no change in the clinical section, because whereas the clerk used to sit with the paper register, now she just sits with the computer and enters the information. In the old days there used to be…but now with [D2] and [D6], it is probably because there are two – the one works and the other goes on, so there are no folders left over for the following day. The next day they start with the previous day's work. Many times in the afternoon they are busy capturing folders from the same day. There is seldom folders that go over into the next day.

HM: You recently moved the ARV-folders to the PHC reception. Has this changed things in the clinic at all, the fact that the folders need to come back to the clinic at the end of the day to be captured?
The only problem we had with that is that the inside-folders are now placed in the day hospital folders and kept at reception. In the past we used to work only with the inside-folder. So if the patient received his medication the folder would immediately go to the clerk for capturing. But now the patient goes to the pharmacy himself, and from the pharmacy, not all the folders are returned to the clinic. So then what happens is, in a month's time, we see on TIER.Net, this is what is good about TIER.Net – he gives me a list at the end of the month, identifying who has not been to the clinic this month. Now we must physically draw those files and then we may see, but 'Joan' was here, maybe 'Joan's' file was at the pharmacy, from us it went to the pharmacy, and from the pharmacy it went to PHC reception. So it did not come to our clinic to be captured. So what is good about TIER.Net, he tells me within a month who has not been. Who is late, then I can go look for those people who were really not here this month, we can trace them.

HM: And is it better now? The system that brings the files back to you?
It is still… I had a talk with them but the head pharmacist is very forgetful. So they have a system where they put all the folders on a trolley, which then goes to reception, and they put all our folders in a box that goes back to the ARV-clinic. So when he's done giving the medication to the client, he forgets where it must go, and he puts the folder on the trolley. How we overcome this problem is to draw the defaulter list every month, and then we physically check the folder – who was here, who was not here. It is a lot of trouble, if you think about [D2]'s workload when [D6] is not here.

HM: Any other challenges you experienced during the implementation process?
The doctors don't always write the information in the clinical stationery properly. We have addressed them about it, but we have a high turnover of doctors, because there are different doctors here every day. And you don't feel like repeating the same story to every doctor every day. So then eventually you stop.

HM: How many patient records were entered during the back-capturing process?
Easily about 3000.

HM: How many data clerks were available to enter those data?
Two full-time clerks.

HM: How long did it take?
[*Thinks for a while*] December 2010 – March 2011.

HM: How did you feel about having TIER.Net implemented in the clinic?
I was willing, because I know electronic things work faster and is more accurate. So that I was very willing for. What worried me is the electronic business crashing, but we have a back-up now. So now I am much more at ease with the system. I don't want to come tomorrow or the day after and then all our information is lost.

HM: What do you think are the challenges of implementing something electronic in a clinic?
If the computer breaks, that will be a challenge for us. And if the system crashes, that will be a problem. If there are power failures, that is also a problem. But physically in the clinic, it won't be a problem for us.

HM: Would you have liked to receive training for TIER.Net?
Yes they must train me as well. If the clerk is not here I have to do it.

HM: Do you think it would have been good if everyone in the clinic received training?
Yes, it would be good. I knew that the register was on, so I didn't have training but I could read what it says, and give out the information in it. But with TIER.Net, a computer, someone needs to show me. I am not that good with technology. [The partner implementer] showed me in December when I had to do the monthly report, and he told me telephonically how to print it. I could do it myself.

HM: And to look up patient details? Was it [D2] who showed you, or did you teach yourself?
No, [D6] showed me how to search. So I can only search and look at the medication and that and that. I cannot look for many things on TIER.Net.

HM: Statement: TIER.Net has improved the data quality of our routine ART Monitoring and Evaluation data.
4 – Agree. More accurate data. You should have seen what that register [the paper register] looked like. [*She shows with her hands how big the pages were. She points on the 'page' to show where the person's name would be and how you would have to go along the narrow row, entering data. If you don't look carefully and follow the line the patient's name is in, you can easily fill in the wrong information by going over into another patient's row. Later when you want to enter new data on a patient, you may find that the data has already been entered, which means another patient's data was entered under the wrong name. Fixing the mistakes are often very difficult or even impossible*]. The registers were big and clumsy and were not user-friendly. When it came to the monthly and quarterly reports it was a nightmare to count.

HM: Statement: TIER.Net has had a positive impact on my work.
5 – Strongly Agree. In the past it took [D2] five days to do the quarterly report. If she hears me now...[*laughs*]. I tell you, five days [*cannot stop laughing*].

HM: Statement: TIER.Net has improved the quality of services we deliver to our clients on ART.
5 – Strongly Agree. Look, if a person is without medication for a month then he is classified as a defaulter. So if I can find him before the month is out, then it brings my defaulter rate down. So if I get the list and I send the PAs to go look for him and the person comes back, then I can easily decrease my defaulter rate.

HM: Does your facility use the data entered in TIER.Net? Yes

HM: How does your facility use the data that is collected through TIER.Net?
We use it for defaulter tracing, and for the monthly and quarterly stats. We also use it to look up patient information. Managers want the stats. And it goes to district and sub-district levels and to the Metropole.

HM: How do you use the monthly stats?
What's important for me now is to see how many patients we started that month. And then we can look at the defaulter rate, what is the defaulter rate for that month, with the visits. That gives me a vague idea of what it looked like.

HM: Do you have any other issues you would like to share on TIER.Net?
None
