# Supplementary material for: Implementation of an Electronic Monitoring and Evaluation System for the Antiretroviral Treatment Programme in the Cape Winelands District, South Africa: A Qualitative Evaluation
Source: PLoS One. 2015 May 12;10(5):e0127223. doi: 10.1371/journal.pone.0127223 (PMC4429075; doi:10.1371/journal.pone.0127223)
Supplement: S2 Information — (PDF) [file pone.0127223.s002.pdf]

Date conducted: 21-02-2012
Date transcribed: 27-02-2012
Interviewer: HM

Interviewee type: F
Interviewee code: F1
Site: Facility C

HM: What is your role in TIER.Net?
I don't physically work with the programme, enter data, etc. but at the end of the day it is my responsibility, because if I sign off that the information on which I am reporting is correct, and there are mistakes, then it comes back to me.

HM: What is your experience with TIER.Net?
With the e-register it is...when [the clerk] gives me the information, it is, because it is done electronically and I double-check, everything is done right. And she does it – she does all her recording every day while she is busy in the clinic. So at the end of the day she has all her information. For me there is no information which goes missing. And then if she, for example if I... we want to correlate anything, for example which patients she must go back to, how many patients were defaulters...then, understand, with the register, she does not physically have to go page through a bunch of registers. Everything is available electronically. For me it is very, really very...our work...it has made it much easier, improved. But, I am dependent on her to give me information.

HM: How did you experience implementation?
The training was organised by Anova and it was basically just the staff who worked in the ARV-clinic [who received training]. So the rest of the clinic's staff was not trained in the e-register. But everyone was trained in the ARV-stationery. But the e-register only the staff who work in the ARV-clinic.

HM: How did implementation impact on the clinic?
No, it did not have an impact at all. Actually it made our lives easier in the sense that it was only the staff who worked in the ARV-clinic who went for training, which is the same as any other normal trainings, so you have to make provision for that. When staff have gone for training then you must...and there is a clinic day then you must make other staff available. And it was only for a few days so it was a normal process, we must just ensure that all the sections are covered. And I also think the doctors accommodated us in the sense that when staff had to go for training they just booked less patients for that time period.

HM: What about the back-capturing?
[The admin clerk] had started here and then we started with the...she started with the e-register [a few months after]. At that stage some of our information went, let's say, missing. The clerk who worked here before her did not...we don't know what happened. All the information was not...she couldn't report to us. She for example, did not transfer [the information] to [the new clerk]. So [the new admin clerk] did not know what was going on and then she literally had to go sit and physically count and rewrite the patients [into the paper register]. It was a lot of work for her. Just after that we began with the e-register and then she could...Do you understand, she already started with it, and so she could just enter all that information [onto TIER.Net].

HM: How did you manage the time she needed to back-capture?
During that time we let [the admin clerk concentrate on the back-capturing], because we realised that it is an extra load on her, and she had to get all the patients on the system. And before [the back-capturing] she was also busy updating the registers with the previous information which just disappeared, and the clerk, I

think it was a temporary clerk...she resigned...not resigned, the [new admin clerk] was appointed to that post and then she...we don't know what went wrong there. Understand, we told [the temporary clerk] to come in and train [the new admin clerk] but she never showed up. So [the new admin clerk] was thrown in the deep end. So we just realised, and that is why more time...we loosened her so that she could go on [to back-capture]. It was important that that work must be done. And later when the process was on the go, [the admin clerk] helped us at reception in the mornings, except on Tuesdays and Thursdays, on some mornings, especially Fridays, just for an hour or so if there was a lot of pressure. Thereafter we let her work on the back-capturing, especially on Fridays.
HM: You felt that it is something important...
Which needs to be done. And she was still new, so she would have to spend extra time on that.

HM: Did you experience a difference in work flow before and after implementation?
Our patients from the ARV-clinic's files are kept separately [in the ARV-clinic], so the patients do not go through reception. They keep their own head count at the ARV-clinic. So the files are filed in the cabinets [in the ARV-clinic]. That process...I don't think there was a lot of hold-up around that. TIER.Net did not make that process faster.

HM: How many patient records were entered during the back-capturing process? More than 200.

HM: How many data clerks were available to enter those data? One admin clerk.

HM: How long did it take? I cannot say with surety.

HM: What do you think makes TIER.Net work in this clinic?
If there are mistakes on the stationery, then [the ARV-nurse] comes back to the PHC sister so that they can fix it. Or they come to me, then I ask the sister to fix it. And we look to see that is has been filled in. That the stationery has been filled in correctly. If I do an HIV-audit for example, we have to audit our files every term. We don't always get to it – every term or every six months – but then we make sure that every sister...if she made mistakes then she must fix it herself … so every now and then mistakes slip through because someone is not doing their job properly.

HM: Were there any challenges during implementation?
Space is an issue, especially on Thursdays when the doctor is in, because then we do not have space for the doctor to work. [The admin clerk] eked out a small corner and you know we are very on top of each other. I get claustrophobic for her sake in that small corner. And now I want to put a door on. She asked me if she could be more isolated, because it is not private – for the patient to sit in that room and everyone is there. It makes the patient fearful. Confidentiality, and my information – the entire world knows my information.

HM: Statement: TIER.Net has improved the data quality of our routine ART Monitoring and Evaluation data.

5 – Strongly agree. Because [the admin clerk] is obligated to enter her information every day, and at the end of the week she gives my stats for me, defaulter lists, who gets medication, how much medication has been dispensed, how many patients have been referred, how many patients have started, how many ARV patients are there altogether, and which patients have started on Bactram. That's the stats. And then as an extra, I also want to know from her how many hours the doctor has worked.

HM: Statement: TIER.Net has had a positive impact on my work.
5 – Strongly agree. Definitely. The information that I need is available much faster, and then it is also not so time-consuming …I can also go back to the last month much faster. I don't have to go search in the files.

HM: Statement: TIER.Net has improved the quality of services we deliver to our clients on ART.
4 – Agree. Yes, because you have all the information available. And the information is also available faster. And because it takes less time to find the information, you have more time to attend to the other needs of the patient. The quality of work is better. The quality of stats is also better. And I can use it.

HM: Does your facility use the data entered in TIER.Net? Yes

HM: How does your facility use the data that is collected through TIER.Net?
We use the data to see which, okay, me as the facility manager, the health promotors, the nursing staff, our counsellors, and then the information goes to the sub-district, to our RMR. And then from there it goes to district. We use the data mostly for reporting. And to look at your defaulters, your co-infections, TB co-infection, and patients who…the referrals. Because we link with home-based care, hospice. We work with them to identify areas for patients that need referral.

HM: Can you see ways of using the data from TIER.Net in the long term, for which you are not already using it?
Yes, to see the impact that HIV has on our area, like "know your epidemic."

HM: What would you change about the TIER.Net system?
Because I don't really work with it I don't feel that I can really say.

HM: Do you have any other issues you would like to share on TIER.Net?
Yes, if more of the staff are trained, and the other clerks as well – the other reception clerk [PHC]. Because at the moment it is only the ARV-clerk who is trained in TIER.Net. And the ARV-sister [must be trained], the sisters who had NIMART training must also be trained in TIER.Net so that they have insight into how it works.
