# Supplementary material for: Implementation of an Electronic Monitoring and Evaluation System for the Antiretroviral Treatment Programme in the Cape Winelands District, South Africa: A Qualitative Evaluation
Source: PLoS One. 2015 May 12;10(5):e0127223. doi: 10.1371/journal.pone.0127223 (PMC4429075; doi:10.1371/journal.pone.0127223)
Supplement: S3 Information — (PDF) [file pone.0127223.s003.pdf]

Date conducted: 20-04-2012
Date transcribed: 01-05-2012
Interviewer: NF

Interviewee type: H
Interviewee code: H2
Site: District/Sub-distrct

NF: What do you do, how long have you been working here?

I am the SAO, information management, and it is basically for the HAST [programme].

NF: What is SAO?

Senior admin officer. I do the HAST, but it is not only HAST. Also the other data that the clinics send.

NF: The RMR?

Yes … going on 9 years [*laughs*] but not ART for 9 years, the other part of HAST. There have been many changes and such. ART only now came over to HAST, something I actually feel is long overdue.

NF: And how do you feel? When you started versus now...?

When I started, HAST was separate and there was a designated person in the building that did it and when that person left, I had to take over and I did not have training so I took it and started interpreting by myself and definitions and so on.

That time versus now… [*thinks*]…I think the quality might have improved a little.

But, it feels like nobody is actually looking at the stuff. It's like it just gets submitted. This is due. There is not really … It has been said that, at the moment the stuff is looked at, but if we look at the data, in depth, then you realise that it has not really been looked at. I have now started; I did not do the ART previously. It's only now since December [2011].

So, I attended the Master training and it helped a lot. And I exported the data myself and started looking at it and it helps a lot when you look at it yourself. And you learn what is not working … which columns … how you need to interpret the data … the gaps … which areas need more work. I did two sub-districts. I sent [my findings] through, but I have not looked at it again… did it improve? I tell you it really takes time to look at it. Because there are a lot of patients, it is a significant amount of time.

NF: How do you receive the data? Per patient or per…?

Monthly we get a report only, a one pager. New patients, so how many remaining in care. Quarterly, we get the dispatch. TIER.Net's dispatch and then I load it [on my computer] and then I send it on [to province]. So it is quite difficult at the end of the quarter. Data needs to be verified. You need to do data quality checks. There are so many things that need to be done at the end of the quarter, reports and the like, so I only get a chance to look afterwards. And then I can only have a look at one sub-district, because there are so many patients. And then only send it back to them and then I will only see a difference next quarter

NF: How do you give feedback?

What I did, because it is patient information…it includes the names of the patients, the patients that I queried I highlighted and then I put a password on the file. So I would send the email and explain why I queried some of the stuff. For example, possible duplicate patients, incorrect dates – they need to check the date – and the like. And then I also tell the [ART Programme Coordinators] if they want the password, they must send an email, then I will mail the password. I cc'd my supervisor for her to see that I am giving feedback. Then I send the spread sheet.

NF: Then you also know when they look at it?

That I do not know.

NF: So you provided feedback to the [ART Programme Coordinators], not the clinics?

We have a new SOP that will be finalised at the end of the month and that will be sent out next month. Then the data and communication flow will change a little, how they report and the like. So the [ART Programme Coordinator]…we are not allowed to contact them directly. We have SAOs, information management in the sub-districts, so the [ART Programme Coordinator], they give the data to info and they will send it to us and we will send it on to province. If there are any queries, I send it back to info and they will filter it down to the [ART Programme Coordinator], who will follow it up with the facility.

NF: Ideal, now they will look at the data more in-depth at sub-district…

They are supposed to. They got an extension on time to look at the data, clean it, so they are supposed to go and look at the data, especially the report that is most important, is the defaulter report, so they need to clean that report before they send it. But, at the moment, they are not looking at it yet. So it is difficult, I don't know, at the moment they concentrate more on TB and not so much on ART. I don't know at the SOP workshop they demonstrated how you interrogate the data, you send it to the Excel spread sheet or you can do it on the programme, but if they went back and implemented it… did it… we do not know.

NF: So they had training, the [ART Programme Coordinator] how to…?

Yes and their clerks and [the partner implementer] is also offering refresher courses per sub-district for both the clerks and even the operational managers of the sites, they are also getting training now.

NF: What is your hope for TIER.Net?

You know, it was something totally new for me. At the moment it is still difficult, because you didn't really get support from the [ART Programme Coordinators], understand? It doesn't help we look at the data but they are not. And at the moment we are not really using the data. We receive a report from province that we use for our M&E. We don't generate the reports to use it, only for quality.

NF: When TIER.Net came was there extra work that was loaded on you or things that were difficult?

My honest opinion, it should have been incorporated a long time ago…

NF: So it was not difficult for you?

Not for me. I don't know about the rest. It was not difficult for me, because HAST, ART is a part of HAST. The ART used to go directly to province and we only got a report. But now it comes here. How can I explain… The people are still not taking responsibility [for ART], although we have integrated. I am talking about at the facility-level, they are not looking at the data. For example, we showed that at one facility there were many patients that was supposed to start ART who have not started yet, so the people are not really looking [at the data]. I find it very interesting, the ART data. Something came up – there are more positive kids under a year or under 2 years [on ART], as compared to how many was reported positive in the PMTCT programme, so now they have to go and investigate. Maybe, it is, how can I explain it .. it might be a capturing mistake … wrong date of birth entered or they have to investigate why is this so, so for me it is interesting. There are many things, for instance the medical terminology and such that we at info are not used to, you see.

NF: It's almost …you learn of these things as you go along?

Yes… but the basics are ok for me. It is when it comes to lab results … it gets a little confusing for me.

NF: All the data that is coming in …PMTCT….RMR...Do you correlate between them?

We have a PMTCT coordinator, so at the moment she is looking at it; she is now investigating why is this so. I can't remember how they picked it up, but we provide her with the data. I will go around and update everybody's computer. They are now starting to look at the data.

NF: How did you find the change process of implementing TIER.Net?

We used to refer to it as the e-register. It wasn't really that difficult. We don't do the capturing, so I would really not be able to capture, but the programme itself was not difficult for me, because we work on ETR and it is exactly the same. My honest opinion is because I am used to what ETR can give me, what I can get out of it, I feel TIER.Net … they can improve it … but this is from an admin perspective not the user's. ETR has checks like 'invalid dates' and this is what I noticed on TIER.Net, the dates don't make sense. A person over 100 years of age... I mean I understand that they are busy to change the programme that way…and then a new version is on its way for which we are going to get training in June, but I am just a little concerned with the new version, the current version exports the patient names to the excel spread sheet, but the new version would not have that. So now you will have to go on folder numbers and it actually worked better when I had both the folder number and the patient's name, because I realised some patients might have two different folder numbers, but it is the same patient at the same facility, so how will I check this if I only have the folder number? They are talking about patient confidentiality… all that, but I feel, we all work with patients.

NF: So does ETR.net show you patient names?

Yes …and it also includes HIV information. So on ETR you can see how many patients are HIV-positive. Like I understand it, they want to integrate the two, because then you have one system where the patient has TB, is positive and now you have TIER.Net where they also have the patient. At the meeting the other day, they spoke about eKAPA…and it sounds like a wonderful programme.

NF: TIER.Net what are the things that it made easier … to work?

You know, I don't work on it as much, only when the dispatches come in, then I will generate the reports, so I don't work on it every day. Because we don't do the capturing part of it I can't tell you anything about that part

NF: But your part, what is working for you?

I am fine with it, but the others was not…I was fine with it … it not like it is a brand new programme … it is the same as ETR with which we are already using … it does not have a very big impact.

NF: How many clinics' reports do you receive...?

[*Shows a piece of paper*] These are the ARV sites that currently send us data as divided per sub-district [*counts… 32*] and they are still busy rolling it out to other facilities …get trained, figure out who is going to capture the data, all that stuff.

NF: I wonder about this thing of 'people to do capturing' …At the start of ART programme there were no clerks, now they're everywhere…

It is my concern as well, a clerk said the other day, they were going to roll it out to new facilities … then I asked him: 'Who is going to do the capturing?' because I mean Anova is not going to be there for ever, Anova only helps with clerks in the beginning … Anova is not going to be there forever, so who is going to capture? Is there a space in that clinic for that person to sit and work, because some of our facilities are small, only two consulting rooms and a reception area… tea room … where will

that person sit to capture? And another thing I asked him: 'what about security?' Computers… what about network points? … that computer needs to be connected to the network .. all the viruses… we know with memory sticks … TIER.Net… like ETR … very sensitive to viruses … for example, the other day, what was difficult for me ... the outside facilities still have the old version, an old version! And I have the newest version. Now I have to import their data and it takes very long. [My PC] showed 'not responding', then you must close the programme and it corrupted my database, luckily I always make back-ups. After each dispatch I make a back-up. And I have to uninstall the programme and reinstall. But for me it is easy, because I know. So for me it is easy, but those are my biggest concerns. Who is going to capture the data if Anova moves out of the facilities? Where will the person sit? Network points? Because some of the facilities do not belong to government, maybe it belongs to a farmer. I don't think they always think about things like that. They just want to implement, implement…

NF: Yes, important stuff…

And we know, I mean, we cannot just employ people, understand? We have budget constraints.

NF: So where are all the extra people coming from at the moment?

Some of the facilities have Anova [staff] and I think the majority we now have in the district … the majority are now permanent. But what has a big impact, for example an Anova clerk working at one of the facilities in the Breede Valley, applied for a government post, and got it. Who are monitoring these things? Because that person must know, you cannot take anybody into that position. The new person needs to know how to capture; the person must know the programme.

NF: That is true, what happened?

Uhm the, we appointed info clerks, to help with the stats for the clinics, so then they took that person [the previous ART clerk], because that person worked at a different facility in the ART clinic on TIER.Net, did capturing. They took him and when he started at the [new] clinic, they took that person to capture the stuff, meaning, the other work the person was supposed to do is delayed, and our clinics gets audited.

NF: Did data quality improve with TIER.Net?

TIER.Net did not improve, the other [data] improved marginally. TIER.Net did not improve ... I do not know what the problem is… Capturing, is TIER being captured? Simple things like the date of birth, captured incorrectly… There are so many things, it just feels like, I mean if I can pick it up here at info, that do not have any medical background, why can't the [ART Programme Coordinators] not also pick it up?

NF: Your other data … does it also come through the [ART Programme Coordinators]?

The other data … same route.

NF: Is that data better …?

I think they concentrate more on TB and not TIER.Net. ETR is being captured by the [ART Programme Coordinator], the green and yellow pages are sent [from the clinic], all the different pages gets sent and gets captured by the HAST clerk.

NF: So they are almost forced to look at it?

TIER.Net gets captured at the ART site, so may… yes [*laughs*] …. I don't know if that could maybe … or maybe they didn't really know how to look at the data. I don't know. Because they, without a SOP, it says they have to look at the data, but now we get dispatches and I wonder: 'but now, did you look at this data for the month, do you understand?'. And it is difficult, because we have due dates, to get the stuff from them. We are not supposed to call sites for outstanding data, because the [ART Programme Coordinator] needs to take the responsibility to get everything from the sites. But now they phone: 'We can't get it [emailed] through.' Then I tell them, 'But it's a report, not a dispatch.' So they also do not always understand. But I get the feeling they are not taking the responsibility, they…like this month I could see clearly; TB is submitted, ART we have queries, this is outstanding, that was late, they are late and they don't care. This is really a problem. So province sends us an email, we need the data for the premier, but… nobody are sent to their managers, the stuff is still late.

NF: Is this worse in some sub-districts?

Yes, [in sub-district C] our data has to be in by the 5th of April for this month, when the new SOP gets implemented, the data has to be at province by the 22nd, but this has not been signed off yet in the circular, so it is still the 7th. So we had to hand-in the data by the 5th of April, they were, I was on leave, they gave us an extension until the 10th of April, when I came back the stuff was still outstanding. Our data was only complete by the 17th of April. [Sub-district E], they put in the effort, they send in their stuff, um [Sub-district A] will also submit, [Sub-district D] no problem, they send in their stuff, um [Sub-district B] … they have difficulty with the correctional services, because those people are on leave and it is difficult to manage them, the correctional services, and then the [ART Programme Coordinator] has to drive out to fetch the stuff, fetch the dispatches.

NF: [Sub-district C] is the problem?

If it is not the PC that has been stolen, then the computer crashed, I don't know what happens. I don't think they realise how important it is to make regular back-ups. At the training they suggested [to back-up] after tea and lunch and before you go home. But I feel, every time you get up from your desk make a back-up, because the electricity, you might be making a cup of tea and the power trips and you lose everything … the file is corrupt or something like that. And that is what they are not doing, they save stuff outside the C drive.

NF: Does TIER.Net give you a better idea at the bottom?

Definitely, definitely. But you have to look deeper at the data. But if they put more user-friendly reports, then it would be easier, especially on our district level, easier if you could just generate a report and see what is going on. And what concerns me a little at the sites, they ask in the SOP, the ART SOP, site visits only every quarter, but I realise there are a lot of sites, but for me – once every quarter

NF: Very little …

Yes.

NF: Something you would change. You mentioned the data quality reports, is there anything else?

TIER.Net, mine freezes a lot, I don't know about the others'. It freezes much too frequently. I don't know why and it is quite frustrating. Every time with a new version they say it will be better, but my programme still freezes a lot.

NF: ….anything else?

At the moment, not a lot, because I do not work on it so often, but with the little that I do, you pick up quite a number of things. I feel, I can… like the dispatches … I am able to assist that person at ground level to do it and even the [ART Programme Coordinators], I even showed province something that they were not aware of – that it does not slow you computer that much and they did not even know that, so um [*laughs*] but I think TIER.Net is a good programme, if it is only used correctly. If it could be strengthened with reports … because it doesn't help we look at it on district and they do not look at it at a lower level. So we can show them some gaps on TIER.Net, but we cannot change it. But I would like it to be more user-friendly in the sense of better reports and so the software can be more stable. Almost every second month there is a new version, and the facilities, the coordinators are not always informed of all the new versions. At the Master training was, we went on the website 'vula', so we now get mails informing us if new versions is coming out, training material or this manual, stuff like that. But people there do not know about it, they don't have access to it, so they are now creating a website where we can actually download training materials and the latest version [of TIER.Net].
