# Supplementary material for: Implementation of an Electronic Monitoring and Evaluation System for the Antiretroviral Treatment Programme in the Cape Winelands District, South Africa: A Qualitative Evaluation
Source: PLoS One. 2015 May 12;10(5):e0127223. doi: 10.1371/journal.pone.0127223 (PMC4429075; doi:10.1371/journal.pone.0127223)
Supplement: S4 Information — (PDF) [file pone.0127223.s004.pdf]

Interview Guide – Data Clerks, Facility Managers, and Nurses

Name of Interviewer		Date	
Location (office/facility, sub-district, district		Time interview started	
		Time interview finished	

Intro script: Good day, my name is [_____] and I would like to discuss your experience with TIER.net (also known as the e-Register). Everything we talk about today will remain anonymous. We will summarise the perspectives of all of the interviews we conduct to learn more about TIER.net and may share de-identified quotes from people we interview in a report or publication. These reports will not include any names or other personal information. Remember that there are no right or wrong answers and we are sincerely interested in your opinions to improve the use of this system and to inform the upcoming national roll-out.

Interviewer instructions:

- [provide informed consent and other background info]
- [Please collect the following information prior to the interview and/or during your greetings/introduction]

Name		Length of time using TIER.net [in months, includes any interaction with system: data entry or report viewing]	
Job title			
Contact telephone (optional)		Contact email (optional)	

**Q1. What is your role in TIER.net (the HIV e-register)? Is this role clear to you?**

**Q2. Think about your work now.** Tell me about your experience with TIER.net.<br>• [probe]: What do you like about it? What do you dislike about it?<br>• [probe]: In terms of your work, what was different before TIER.net?

Interviewer Script: I'll now make a statement. I'd like you to tell me if you Strongly Disagree, Disagree, are Neutral/Unsure, Agree, or Strongly Agree with the statement. Please feel free to explain your answers. I will ask follow-up questions depending on your responses. I will share a few more statements like this during the interview

**Q3. The process of TIER.net implementation was very difficult.**<br>[interviewer, please clearly circle the answer]				
1	2	3	4	5
Strongly Disagree	Disagree	Neutral/Unsure	Agree	Strongly agree
☐	Click here, if respondent does not know.			
[Record explanation of answer below.]<br>• [If Agree, or Strongly Agree, probe] How was it difficult? What aspects were difficult? Provide examples if possible.<br>• [If Disagree, or Strongly Disagree] What made it easy? What aspects were easy? Please provide examples.<br>• [If neutral] Please tell me why you say that.				

**Q4. Tell me more about the process of implementation.**<br>[probe]:<br>• Please explain the process.<br>• How long did it take?<br>• What worked?<br>• What did not work?<br>• The back-capturing process.<br>• How did the implementation impact on work-flow in the clinic?

**Q5. How many patients were entered during the back-capturing process?**	
**Q6. How many data clerks were available to enter those data?**<br>[probe]:<br>• Did you need to recruit additional staff (permanent paid, part-time paid, or volunteers)?	
**Q7. How long did it take?**	

**Q8. Tell me about your work experience before, during, and after TIER.net was implemented.**<br>[probe]:<br>• What were the challenges you had with the system during this process?<br>• Software or database issues?<br>• Any hardware (not the database, but issues with the computer) problems?

**Q9. What do you think makes TIER.net work in this clinic?**<br>[probe]:<br>• Staff receptivity<br>• Challenges of implementing an electronic programme such as TIER.net<br>• Staff ability<br>• Training and support<br>• Availability of resources (time, equipment, and space)<br>• Other factors

Q10. **TIER.net has improved the quality of our routine ART Monitoring and Evaluation data.** [please clearly circle the answer]<br>- **Ask data clerks about the accuracy of data (more accurate than the paper-register)**				
1	2	3	4	5
Strongly Disagree	Disagree	Neutral/Unsure	Agree	Strongly agree
☐	Check this box, if respondent does not know.			
[Record explanation of answer below.]<br>• [If Agree, or Strongly Agree, probe] How has TIER.net improved data quality? Why do you think that is?<br>• [If Disagree, or Strongly Disagree] How has TIER.net reduced data quality? Why do you think that is?<br>• [If neutral] Please tell me why you say that.				

Q11. **TIER.net has had a positive impact on my work.**<br>[interviewer, please clearly circle the answer]				
1	2	3	4	5
Strongly Disagree	Disagree	Neutral/Unsure	Agree	Strongly agree
☐	Check this box, if respondent does not know.			
[Record explanation of answer below.]<br>• [If Agree, or Strongly Agree, probe] How has it had a positive impact on your work? Provide examples if possible.<br>• [If Disagree, or Strongly Disagree] How has it had a negative impact on your work? Please provide examples.<br>• [If neutral] Please tell me why you say that.				

Q12. **TIER.net has improved the quality of services we deliver to our clients on ART.**<br>[interviewer, please clearly circle the answer]				
1	2	3	4	5
Strongly Disagree	Disagree	Neutral/Unsure	Agree	Strongly agree
☐	Click here, if respondent does not know.			
[Record explanation of answer below.]<br>• [If Agree, or Strongly Agree, probe] How? Provide examples if possible.<br>• [If Disagree, or Strongly Disagree] How? Please provide examples.<br>• [If neutral] Please tell me why you say that.				

Interviewer Script: I'll now ask a few questions about data use and the future of TIER.net.

Q13. **Does your facility use the data entered in TIER.net?**	☐ Yes	☐No

Q14. How does your facility use the data that is collected through TIER.net?
[probe]:<br>• Who uses it?<br>• What about reports?<br>• [If not mentioned, probe on use of the following: monthly, quarterly, missed appointment early & late, and defaulter reports]<br>• What resources would be needed in order to make better use of the data?

Q15. Can you see ways of using the data from TIER.net in the long term/future, for which you are not already using it?
[probe]:<br>• What about Tracking?<br>• Adherence?<br>• Linking HIV testing and pre-ART clients to treatment?

Interviewer Script: For the following items please indicate if TIER.net has had a positive, negative, or no impact at all on the following items [mark an X in the appropriate column, if unknown indicate in explanation box]. Feel free to include why and how.

**Q16. What has been the impact of TIER.net on…**				
**Item**	**Pos**	**Neg**	**None**	**Explanation**
**a.** Quality of clinical stationery				
**b.** Data entry workload				
**c.** Amount of time entering data				
**d.** Patient clinic flow				
**e.** Patient wait time				
**f.** Pharmacy management				
**g.** Stock ordering (e.g. swabs, needles etc.)				
**h.** Human resources management (work load/task-shifting)				
**i.** Programme planning				
**j.** Reporting into information systems				

Interviewer Script: We're just about done; there are just a few final questions.

**Q17. What would you change about the TIER.net system?**

**Q18. Do you have any other issues you would like to share on TIER.net?**

Interviewer Script: Thank you very much for your time and opinions. The results of this study should be available soon. Prior to finalisation, a draft report will be made available for review and feedback.
